# Footwear Identity and Postoperative Experiences of White-Collar Women After Hallux Valgus Surgery: A Qualitative Study

**DOI:** 10.3390/healthcare14040547

**Published:** 2026-02-22

**Authors:** Mehmet Yiğit Gökmen, Mesut Uluöz, Mehmet Maden, Özhan Pazarcı, Talha Tepeoğlu, Osman Çiloğlu

**Affiliations:** 1 Department of Orthopaedics and Traumatology, Faculty of Medicine, Çanakkale Onsekiz Mart University, 17110 Çanakkale, Türkiye; 2 Department of Orthopaedics and Traumatology, University of Health Sciences, Adana City Training and Research Hospital, 01230 Adana, Türkiye; mesutuluoz@hotmail.com (M.U.); dr.pazarci@gmail.com (Ö.P.); osmanciloglu@gmail.com (O.Ç.); 3 Department of Orthopaedics and Traumatology, İzmir Atatürk Training and Research Hospital, 35360 İzmir, Türkiye; mhmtmdn@gmail.com; 4 Department of Orthopaedics and Traumatology, Ömer Halisdemir University Training and Research Hospital, 51100 Niğde, Türkiye; talhatepeoglu@gmail.com

**Keywords:** hallux valgus, osteotomy, internal fixation device, metatarsal deformities, qualitative research, white-collar workers, footwear, patient-reported outcomes

## Abstract

**Background:** Hallux valgus affects footwear tolerance, body image, and social participation, particularly among white-collar women who adhere to formal dress codes. While clinical outcomes of hallux valgus surgery are well described, little is known about how women in office-based occupations experience postoperative recovery. This study explored the lived experiences of women at least 12 months after surgical correction of mild-to-moderate hallux valgus using distal first-metatarsal osteotomy with adjustable intramedullary T-plate fixation. **Methods:** A qualitative interpretivist approach was employed. Semi-structured interviews were conducted with purposively selected Turkish-speaking white-collar women who underwent surgery between January 2021 and January 2024. All had ≥12 months of follow-up. Interviews were transcribed verbatim and analyzed using reflexive thematic analysis guided by Consolidated Criteria for Reporting Qualitative Research (COREQ) principles. Trustworthiness was supported through member checking, an audit trail, negative case analysis, and peer debriefing. Data saturation was reached at 27 interviews. **Results:** Twenty-seven women (mean age 43.04 ± 4.66 years) participated. Six themes emerged: (1) expectations and motivations; (2) postoperative physical experience; (3) aesthetic perception; (4) psychological responses; (5) social and domestic support; and (6) footwear identity and adaptation. Participants described meaningful gains in comfort, confidence, and mobility. The ability to choose footwear freely, rather than endure pain, was central to their sense of recovery. Improvements in self-image and ease in professional and social settings were also emphasized. **Conclusions:** Across six interrelated themes, white-collar women described postoperative recovery as a multidimensional process encompassing footwear autonomy, body image, occupational confidence, physical experience, psychological responses, and social support. These findings highlight the importance of incorporating footwear expectations and workplace needs into preoperative counselling and postoperative care.

## 1. Introduction

Hallux valgus (HV) is one of the most common forefoot deformities and a frequent indication for elective foot surgery. Recent meta-analyses estimate a global pooled prevalence of roughly one in five adults, with consistently higher rates in women than men, underscoring both the scale and sex disparity of the condition [1].

In addition to intrinsic and familial factors, footwear practices, particularly narrow toe boxes and elevated heels, have been repeatedly implicated in symptom burden and disease progression, linking everyday footwear choices to biomechanical stressors that reinforce visible deformities and restrict daily activities [2,3,4].

Beyond local pain at the first metatarsophalangeal joint, HV is associated with decrements in foot-specific and general health–related quality of life in adults and older adults. Patients report functional restriction, difficulty selecting tolerable footwear, and social self-consciousness related to foot appearance, demonstrating that the impact of HV extends well beyond radiographic angles and clinical assessments [5,6].

Surgical correction aims to realign the forefoot and relieve pain. However, substantial heterogeneity in procedures (e.g., distal and shaft osteotomies, Lapidus, minimally invasive variants) and variability in outcome reporting complicate comparisons across studies [7].

While modern series document substantial gains on patient-reported measures, the literature also notes a meaningful minority with residual dissatisfaction, highlighting that symptom relief, shoe tolerance, cosmesis, and expectations can diverge [8,9,10].

Qualitative evidence centered on patients’ perspectives remains relatively sparse for HV, despite the volume of quantitative outcome studies. Emerging studies have examined preoperative expectations and postoperative recovery narratives; however, detailed accounts of how occupational demands and social environments shape postoperative adaptation are still limited [11,12,13].

This gap is particularly relevant for office-based “white-collar” workers, for whom dress codes and client-facing roles can create persistent tension between professional appearance (e.g., formal or heeled shoes) and postoperative comfort. In many professional settings, including those common in Türkiye, such as education, administration, finance, law, engineering, and healthcare management, business-casual or formal footwear is routinely expected. Recent discussions on occupational health have highlighted the risks of mandatory high-heel requirements, emphasizing the need to consider workplace footwear norms when evaluating musculoskeletal outcomes [14,15].

Against this backdrop, the present interpretivist qualitative study examines the lived experience of white-collar women at least 12 months after surgical correction of mild-to-moderate hallux valgus using a distal first-metatarsal osteotomy with fixation by an adjustable intramedullary T-plate [16]. This study was guided by an analytic proposition that postoperative success in this population would primarily be achieved through restored footwear autonomy, body image, and occupational confidence, rather than radiographic or purely clinical outcomes alone. We aimed to examine the intersection of physical recovery, footwear identity and choice, body image, and psychosocial adaptation in this occupational group, to inform patient-centered counseling, footwear guidance, and return-to-work planning.

## 2. Materials and Methods

### 2.1. Ethical Approval

Ethical approval for this study was obtained from the Adana City Training and Research Hospital Clinical Research Ethics Committee on 21 August 2025 (meeting no. 16, decision no. 704). All procedures were conducted in accordance with the principles outlined in the Declaration of Helsinki. Participants received both written and verbal information about the study and their rights, including the right to withdraw without consequences, and written informed consent, as well as separate permission for audio recording, were obtained from all participants. The sampling frame comprised patients who underwent surgery between January 2021 and January 2024. All screening, invitations, and interviews were initiated only after ethics approval was obtained, and each participant had undergone surgery at least 12 months prior to the interview.

### 2.2. Research Design, Setting, and Participants

This qualitative study was conducted within the interpretive paradigm, acknowledging that experiences are socially constructed and subjectively perceived. Semi-structured, in-depth interviews were conducted to explore the lived experiences of individuals at least 12 months post-surgical correction of mild-to-moderate hallux valgus using adjustable intramedullary T-plate fixation (Supplementary Material S1, Surgical Technique). The study adhered to the Consolidated Criteria for Reporting Qualitative Research (COREQ) guidelines [17].

The semi-structured interview guide (Supplementary Material S2) was developed by MYG and MU based on the study aims, clinical experience in the care of hallux valgus, and the qualitative literature on patient expectations and postoperative adaptation. A provisional version was drafted with open-ended questions and optional probes to ensure coverage of footwear identity and choice, body image, psychosocial adjustment, and workplace-related demands. Pilot testing was conducted with a small number of eligible postoperative patients to evaluate clarity, flow, and acceptability of the questions; feedback was used to rephrase ambiguous items, adjust prompts, and refine the order of topics. The final guide (Supplementary Material S2) retained the same thematic coverage while improving comprehensibility and conversational flow.

The study was conducted at the Orthopaedics and Traumatology outpatient clinic of a tertiary orthopaedic center in Adana, Türkiye. Eligible participants were retrospectively identified from hospital records of patients who underwent unilateral distal first-metatarsal osteotomy with adjustable intramedullary T-plate fixation between January 2021 and January 2024. The same surgical team performed all procedures, ensuring consistency in operative technique.

To refine analytic focus, inclusion was restricted to white-collar employment, and all participants were women, operationalized as office-based or professional roles predominantly involving desk/meeting duties and formal or business-casual footwear requirements. Purposive maximum-variation sampling was used to ensure diversity in age, occupational sector, and minor postoperative outcomes. Screening and recruitment were performed by two orthopedic surgeons (MYG and MU) from the research team. Inclusion criteria: age ≥ 18 years; female sex; able to communicate in Turkish and provide informed consent; primary surgical correction of hallux valgus with adjustable intramedullary T-plate fixation; ≥12 months postoperative follow-up; availability of clinical and radiographic data, and current or recent white-collar woman employment. Exclusion criteria: manual/service or shift-based occupations requiring safety footwear or prolonged standing; revision HV surgery or additional midfoot/hindfoot procedures; major coexisting forefoot deformities; cognitive or psychiatric impairment precluding informed participation; incomplete follow-up records.

A total of 27 participants were recruited. The research team judged that data saturation had been reached at the 24th interview; three additional interviews were conducted to confirm that no new themes emerged.

### 2.3. Data Collection and Analysis

The semi-structured interview guide (Supplementary Material S2, Form SA) was co-developed by MYG and MU and subsequently refined through contributions from researchers (ÖP and TT) following pilot testing. The initial interviews were conducted in the last week of August 2025. Two researchers conducted interviews (ÖP and TT). Each session lasted 30–75 min, was conducted in Turkish, and was audio-recorded with the participant’s consent. Depending on participants’ preferences, interviews were conducted face-to-face in a quiet consultation room or via secure videoconferencing, with field notes used to capture nonverbal and contextual cues. After preliminary theme development, additional member-checking interviews were conducted approximately two weeks later, in mid-September 2025, with selected participants to confirm the accuracy of interpretations. Questions evaluating hospital service quality were excluded to maintain focus on lived experiences. For manuscript preparation, transcripts were professionally translated into English and back-translated into Turkish, with discrepancies resolved by consensus among the translators and the research team.

All interviews were transcribed verbatim and analyzed using reflexive thematic analysis. Two researchers (MM, OÇ) independently conducted open coding, guided by Saldaña’s Coding Manual [18]. They synthesized their work into a shared codebook, which was iteratively refined and applied to the entire dataset. Codes were organized into candidate subthemes and themes, which were then reviewed for coherence and finalized through discussion. Trustworthiness was ensured through several strategies, including member checking (Supplementary Material S2, Form SB) whereby brief theme summaries were returned to participants for confirmation and feedback; negative case analysis to refine theme boundaries; maintenance of a detailed audit trail documenting coding decisions and analytic steps; provision of thick description of occupational contexts such as formal footwear norms, client-facing duties, and graded return-to-work processes; and researcher reflexive memos supported by periodic peer debriefing. Quotations presented in the Results are anonymized and labeled by participant number.

## 3. Results

Twenty-seven participants (all women; mean age 43.04 ± 4.66; range 35–52 years) participated in this study. All participants were white-collar women who had undergone surgical correction for mild-to-moderate hallux valgus using adjustable intramedullary T-plate fixation and had completed at least 12 months of postoperative follow-up. The demographic and occupational characteristics of the participants are presented in Table 1.

Thematic saturation was achieved after the 24th interview; three additional interviews were conducted to ensure analytic depth, yielding a final sample of 27 participants. Reflexive thematic analysis identified six overarching themes and 19 subthemes describing patients’ lived experiences, as summarized in Table 2.

### 3.1. Expectations and Motivations Before Surgery

Participants described the preoperative period as an extended negotiation between functional limitations and social expectations. Intensifying discomfort and narrowing footwear options were closely linked to heightened social awareness of foot appearance, which, in turn, shaped cosmetic dissatisfaction and the decision to seek surgery. Help-seeking was typically initiated after the failure of conservative strategies (wider lasts, insoles, taping, event-based planning), and the final decision was framed as a threshold moment: when social and functional costs outweighed fears about surgery and recovery.

#### 3.1.1. Foot-Related Physical and Cosmetic Complaints

Beyond localized bunion pain, participants reported secondary problems such as pressure marks, corns, and transfer pain, emphasizing that hallux valgus was experienced as both a physical burden and a visible deformity associated with social stigma.

“…Even when it wasn’t actively hurting, I couldn’t stand the way it looked. In family photos or at social gatherings, I would go out of my way to hide my feet. I even cropped them out of pictures because the bump embarrassed me so much.”

(Participant 3)

“…By the afternoon, the ball of my foot would be burning so badly that I changed the way I walked just to take the pressure off. It wasn’t just physical pain; it made me feel awkward in public because people could see I was limping or avoiding steps.”

(Participant 4)

#### 3.1.2. Shoes, Discomfort, and Social Appearance Pressure

Workplace dress codes and formal events were described as arenas in which participants were compelled to negotiate between appearance norms and bodily comfort. They reported adopting compensatory strategies such as carrying multiple pairs of shoes, strategically leaving events early, or avoiding social gatherings altogether, reflecting how footwear became a site of both social performance and personal compromise.

“…I always carried two pairs of shoes with me, one pair of narrow formal shoes that looked right for client meetings, and another pair of comfortable flats I could switch into the moment the pain became unbearable. It felt like I was planning my entire day around which shoes I could tolerate and when.”

(Participant 8)

“…Every summer, I dreaded the thought of wearing sandals. Vacations or holidays that were supposed to feel carefree became stressful because I was worried about people noticing my feet. Sometimes I even planned trips or social events around the idea of hiding my bunion, which took away the joy of those moments.”

(Participant 21)

#### 3.1.3. Decision-Making and Expectations from Surgery

Peer experiences, clinician advice, and online resources informed surgical decisions. Expectations centered on regaining physical comfort and mobility, while also achieving a normalized foot silhouette to reduce self-consciousness. Anticipated risks included prolonged recovery, visible scars, and the possibility of over- or undercorrection, underscoring the dual emphasis on functional and aesthetic outcomes.

“I waited for years before finally deciding on surgery. At some point, I realized that my entire life had started to shrink around my shoes, what I could wear, where I could go, and even how long I stayed at events. I kept postponing the decision, hoping I could manage with insoles or wider shoes, but eventually it became clear that my world was getting smaller, and I didn’t want to keep living that way.”

(Participant 17)

“…For me, the first priority was comfort. I just wanted to walk without constant pain. But at the same time, I was also hoping for a foot I wouldn’t feel the need to hide. I didn’t need perfection, but I wanted to be able to wear normal shoes again without feeling embarrassed every time I looked down.”

(Participant 5)

### 3.2. Postoperative Physical Experience

Recovery trajectories were described as dynamic, beginning with early swelling and pain management and progressing toward restored mobility and expanded footwear choices. Participants often measured recovery not by time elapsed but by functional milestones, such as attending a social event or resuming a full workday in formal shoes.

#### 3.2.1. Pain Course, Swelling, Weight-Bearing, and Wound Care

Pain was typically most intense during the first one to two weeks, followed by a gradual reduction. Swelling required consistent management, and structured guidance regarding protected weight-bearing and wound care reduced uncertainty and prevented overexertion. Scars were a frequent source of concern, but adaptive strategies such as silicone pads provided reassurance and facilitated mobility.

“…By the second week, I finally felt like I had turned a corner. The first days were rough; every step reminded me of the surgery, and I wondered if it would ever get better. But around week two, the pain stopped dominating every thought, and I started to feel like I could focus on normal daily things again rather than just coping with discomfort.”

(Participant 2)

“…The scar was the part I didn’t expect to bother me so much. With stiff shoes, it stayed tender for months, almost like a constant reminder of the operation. Using silicone pads and switching to softer shoes made a huge difference. Once I figured out those tricks, I stopped worrying as much and felt more confident about moving around.”

(Participant 23)

#### 3.2.2. Changes in Mobility and Shoe Choices

Participants highlighted improved stamina, better stair negotiation, and a shift toward footwear options rather than restrictions. The ability to choose shoes freely, rather than enduring discomfort, was repeatedly framed as a tangible marker of surgical success.

“…Now I can walk the entire school corridor without constantly scanning for a chair to sit down on. Before surgery, I would map out where I could stop and rest, almost like planning a route with pit stops. These days, I move more freely, and it feels like a weight has been lifted. I’m no longer calculating every step.”

(Participant 19)

“…Some of my old shoes still don’t work for me, especially the tighter pairs, but I’ve replaced them with softer brands that don’t pinch or rub. At first, I thought this meant giving up on style, but I’ve actually found options that look good and feel good. It’s liberating to choose shoes I like, instead of just tolerating the least painful pair”

(Participant 11)

#### 3.2.3. New Physical Routines (Footwear Strategies, Toe Exercises)

Daily practices such as toe stretches, shoe rotation, and icing regimens were integrated as self-management strategies to enhance agency and minimize setbacks. These routines were described as small but cumulative contributors to long-term adaptation.

“…I figured out that rotating two pairs of shoes during the week really helps. If I wear the same pair every day, pressure points start to build, and I end up with painful hotspots. By alternating between two comfortable pairs, I can keep my feet from flaring up and feel more in control of my routine.”

(Participant 7)

“…Five minutes of stretching my toes each morning has become a habit. If I skip it, the joint feels stiff for the rest of the day. It doesn’t take long, but it sets the tone. I feel looser and more confident moving around when I start the day this way.”

(Participant 20)

### 3.3. Aesthetic Perception and Body Image

Most participants reported satisfaction with improved foot alignment, which facilitated social interaction and reduced self-consciousness. Perceived aesthetic success did not require perfection; rather, the ability to appear “ordinary” was sufficient to restore confidence in both public and private contexts.

#### 3.3.1. Perception of Foot Appearance After Surgery

Participants described a process of re-identifying with their feet, emphasizing that improved alignment helped restore a sense of bodily integrity. Residual scars or swelling were acknowledged but considered minor in relation to the overall gain in silhouette normalization.

“…The bump is gone, and the line of my foot looks clean again. I even notice it when I’m just wearing socks; it feels like my foot has returned to being part of me, rather than something I was ashamed of. For years, I avoided looking at it, but now I catch myself checking in the mirror with relief.”

(Participant 1)

“…It finally looks like my foot, not something I need to hide. Before surgery, I was always conscious of the bunion. Now I don’t think twice about it. I can just be myself without that constant awareness.”

(Participant 13)

#### 3.3.2. Comfort in Public Settings

Situations that previously triggered self-consciousness, such as workplace meetings, social gatherings, and formal events, became neutral or even positive after surgery. Participants described feeling able to participate spontaneously without the constant awareness of their feet that had shaped their preoperative experiences.

“…I no longer scan the room looking for a spot where I can keep my shoes on. Before, I would be tense the whole time, trying to avoid situations where I had to take them off. Now, I don’t feel that anxiety anymore. I just joined in without overthinking.”

(Participant 22)

“…I wore open-toe sandals at a wedding for the first time in years, and I completely forgot about them all night. In the past, I would have been so focused on hiding my feet or worrying about how they looked that I couldn’t enjoy myself. This time, never once did I think about my bunion; it was such a relief.”

(Participant 26)

#### 3.3.3. Confidence, Embarrassment, and Identity-Related Shifts

Participants frequently linked improved appearance with broader shifts in self-presentation and psychosocial identity. They reported increased confidence, improved posture, and a greater willingness to engage in professional and social interactions, indicating that aesthetic gains had a cascading effect on their identity and emotional well-being.

“…The surgery changed how I walk into a room. Before, I would come in quietly and try not to draw attention, always aware of my feet and the way I moved. Now I don’t think about hiding anymore, I feel more confident, almost like people see me first rather than my bunion. That shift has changed how I carry myself at work and in social life.”

(Participant 10)

“…It sounds simple, but I stand taller now. I used to hunch a little, almost subconsciously, because I didn’t want anyone looking down and noticing my feet. After surgery, that self-consciousness faded, and I realized my posture and even my mood improved. I feel lighter and more positive when I walk into everyday situations.”

(Participant 4)

### 3.4. Psychological Process and Emotional Response

Emotional trajectories evolved from early apprehension toward cautious optimism. Gains in mobility and appearance were described as reinforcing reassurance, while persistent concerns about recurrence or complications reflected an ongoing negotiation between hope and vigilance.

#### 3.4.1. Fear of Recurrence or Complications

A minority of participants expressed ongoing anxiety about recurrence, hardware irritation, or incomplete correction. These concerns were managed through gradual exposure to activities and reassurance from follow-up consultations, highlighting the importance of clinician feedback in psychological recovery.

“…I still find myself checking the alignment in the mirror, almost like a ritual. It’s my way of staying calm and convincing myself that everything is still in place. Even though I know the surgery was successful, that little voice in my head keeps asking, ‘What if it comes back?’ Looking and seeing that my toe is still straight helps me relax.”

(Participant 15)

“In the early weeks, I constantly worried about doing too much too soon, like walking too far or putting on the wrong shoes. Every new activity felt like a risk. But the regular check-ups reassured me. Hearing from the surgeon that my progress was on track gave me the confidence to slowly test myself without that nagging fear.”

(Participant 24)

#### 3.4.2. Satisfaction vs. Disappointment

High satisfaction was reported when functional outcomes were achieved, even when cosmetic expectations were only partially met. Residual stiffness or scar sensitivity were noted as sources of disappointment but were generally outweighed by the benefits of reduced pain and increased mobility.

“…It’s not perfect, but it’s miles better than before. The way I measure success is by how little I think about my foot now. Before, it was always on my mind, every step, every shoe choice. Now, days can go by without me noticing it, and that in itself feels like freedom.”

(Participant 6)

“The scar turned out to be more noticeable than I expected, and sometimes it still gets irritated with stiff shoes. But when I compare that small inconvenience to the relief I feel in daily life, being able to walk comfortably, stand longer, or go out without planning every detail, it’s a trade-off I can live with”

(Participant 18)

#### 3.4.3. Emotional Adaptation and Motivation for Change

Recovery was framed as a catalyst for broader lifestyle changes, including weight management, exercise adherence, and more balanced routines. These adaptations reinforced perceptions of resilience and long-term well-being.

“This whole process reminded me that small habits really do add up. Doing my stretches, choosing the right shoes, and maintaining a healthy weight, none of these things seemed huge on their own, but together they made a real difference in how I felt. It made me more disciplined in other areas of my life, too.”

(Participant 9)

“…Seeing the progress after surgery made me more consistent, not just with exercise but also with rest. I realized that balance matters, pushing myself when needed but also respecting recovery. That mindset has carried over into my daily life, and I feel stronger for it.”

(Participant 25)

### 3.5. Social and Domestic Support

Family, partner, and workplace responses were described as critical determinants of both the pace and quality of recovery. Practical support during the early postoperative period and ongoing validation of cosmetic outcomes were considered essential for sustaining motivation.

#### 3.5.1. Family Encouragement or Discouragement Pre-/Post-Op

Supportive family members legitimized help-seeking and facilitated adherence to rehabilitation, whereas dismissive or skeptical attitudes delayed surgical decisions and generated feelings of invalidation.

“…My sister had already gone through the surgery, so she knew exactly what I was dealing with. She coached me through that first month, reminding me when to ice, how to elevate, and reassuring me that the swelling was a normal part of the process. Having her guidance made me feel less alone and more confident that I could handle the recovery.”

(Participant 16)

“…Some relatives brushed it off and called it ‘just cosmetic.’ That really stung, because for me it wasn’t about vanity, it was about pain and not being able to live normally. Their comments made me second-guess my decision at first, but eventually I realized they didn’t understand how much it affected my daily life.”

(Participant 12)

#### 3.5.2. Impact on Household Roles and Work Life

Temporary redistribution of domestic responsibilities was common, and employer flexibility eased reintegration into work. In professions requiring prolonged standing, staged returns and footwear accommodations were crucial in enabling sustainable recovery.

“…Taking time off was financially difficult, but I knew I needed it. In those first weeks, I couldn’t cook, clean, or do the usual chores, and my family had to pick up the slack. It wasn’t easy, but it reminded me how important support is during recovery.”

(Participant 14)

“…I had to plan my teaching schedule around breaks where I could sit down. At first, it felt like I was compromising my job, but gradually my stamina caught up. By pacing myself and accepting those pauses, I was able to keep working without burning out or risking a setback.”

(Participant 25)

#### 3.5.3. Partner/Spouse Opinions

Recognition by partners of both functional and aesthetic improvements reinforced patient confidence. Conversely, minimization of cosmetic concerns created ambivalence, underlining the relational dimension of recovery.

“…At first, my husband thought I was just doing this for looks, and he didn’t really understand how much pain I was in. After the surgery, he finally admitted that the change wasn’t only about appearance, but also about the way I moved. He noticed that I walked differently, with less hesitation, and that recognition meant a lot to me. It made me feel validated in my decision.”

(Participant 11)

“My husband backed me from day one. He kept encouraging me through the painful early weeks and reminded me to do my exercises when I didn’t feel like it. Having her support kept me on track, both physically and emotionally. It made the whole recovery process feel less overwhelming.”

(Participant 20)

### 3.6. Footwear Identity and Adaptation Strategies

Footwear was reframed as a domain of regained autonomy, where participants developed individualized strategies to balance style, comfort, and social expectations.

#### 3.6.1. Ability to Wear Preferred Shoes

Participants described a redefined notion of “preferred” footwear, shifting from rigid ideals to practical but aesthetically acceptable choices. The endpoint was the restoration of agency, being able to select footwear based on preference rather than necessity.

“…Heels are back in my life, but only for a few hours at a time; that’s my rule now. I’ll wear them for a dinner, a wedding, or an important meeting, but I don’t push it beyond that. Before surgery, even a short event in heels felt impossible. Now I can choose when to wear them without paying for it the next day.”

(Participant 15)

“I have fewer pairs of shoes now, but all of them actually work for me. I stopped clinging to the ones that looked nice but hurt to wear. Instead, I invested in shoes that are both comfortable and presentable, and that shift has been freeing. Every pair in my closet now feels like an option rather than a compromise.”

(Participant 17)

#### 3.6.2. Comfort Versus Appearance Trade-Offs

Footwear decisions were guided by explicit rules regarding event duration, walking distance, and surface conditions, demonstrating deliberate strategies to avoid discomfort while maintaining social participation.

“…For long workdays or conferences, I always go with wider flats because I know I’ll be on my feet for hours. But if it’s just a short dinner or a special occasion, I let myself risk a small heel. Having those rules makes me feel in control instead of anxious about what the night will bring.”

(Participant 12)

“If I know there’s going to be a lot of walking, there’s no debate, I choose soft soles. It’s not worth the pain just to look a certain way. I’ve learned that comfort actually gives me more confidence because I can focus on the event instead of worrying about my feet.”

(Participant 3)

#### 3.6.3. Social Expectations Around Footwear

Participants emphasized that professional contexts exerted pressure to conform to visible footwear norms. Postoperatively, they reported regaining a sense of presentability without compromising comfort, which enabled greater ease in their occupational and social roles.

“…In my office, shoes are noticed. Before surgery, I felt like I had to apologize for wearing comfortable pairs that didn’t quite fit the dress code. Now I don’t feel the need to explain myself; I can wear shoes that look professional and still feel comfortable. That’s a huge relief.”

(Participant 2)

“Standing in front of a class used to make me self-conscious because my feet hurt and my shoes never felt right. Now I feel put-together and pain-free, which lets me focus on teaching instead of thinking about how I look or whether I’ll make it through the day.”

(Participant 1)

#### 3.6.4. Adaptation via Insoles, Brands, and New Habits

Adaptive practices, such as alternating shoes, using targeted insoles, and shopping in the afternoon, were incorporated into daily routines. These practices were perceived as empowering tools that sustained comfort and prolonged the benefits of surgery.

“…Switching to shoes with wider lasts and adding a metatarsal pad solved the hotspots that used to bother me daily. It took some trial and error, but once I figured it out, I felt like I had cracked the code for keeping my feet comfortable without sacrificing style.”

(Participant 23)

“Trying shoes on in the afternoon made a real difference. Before, I’d buy them in the morning, and they felt fine, but by evening, my feet were swollen, and the shoes were unbearable. Now I only shop later in the day, and the fit I get is the one that actually lasts.”

(Participant 26)

## 4. Discussion

This study characterizes the lived experience of white-collar women at least 12 months after surgical correction of mild-to-moderate hallux valgus using a distal first-metatarsal osteotomy with fixation by an adjustable intramedullary T-plate. Participants described a preoperative period characterized by ongoing negotiation between physical limitations and social expectations, followed by a recovery process in which early pain and swelling gave way to graded mobility and, critically, to a redefinition of footwear identity, shifting from enduring uncomfortable shoes to exercising choice. These findings extend the limited qualitative work in forefoot surgery by making occupational context explicit and by detailing how expectations, satisfaction, and adaptation unfold when professional appearance norms intersect with comfort goals.

The salience of footwear requirements in white-collar roles helps explain why body image and shoe tolerance emerged as coequal targets alongside pain relief. Beyond surgical contexts, public health evidence has suggested links between narrow toe boxes and heel elevation with hallux valgus, lower limb pain, and increased injury risk. Recent policy discussions have questioned the safety of mandatory heeled footwear, underscoring the importance of footwear norms for musculoskeletal outcomes [19,20]. Consistent with these perspectives, participants in this study often adopted a “selective return” to heeled footwear, with limited duration and event-specific use, mirroring observations that some patients resume heels only if the preoperative frequency and height can be matched. In contrast, others reject them entirely (“all-or-none” behavior) [21]. Such choices highlight how the social visibility of footwear in professional settings can refract the perceived value of surgery through everyday shoe decisions

Persistent heterogeneity in operative procedures and reporting standards complicates cross-study comparisons and may obscure why a subset of patients remain dissatisfied despite radiographic success. Comparative reviews indicate that commonly used techniques (e.g., distal chevron, shaft osteotomies, Lapidus, and minimally invasive variants) achieve meaningful corrections; however, longer-term syntheses reveal notable recurrence, reflecting variation in correction philosophy and follow-up definitions [22,23,24]. Against this backdrop, the qualitative accounts in our study suggest that patients define success not only through alignment metrics but also through effortless shoe selection, social ease, and reduced vigilance regarding recurrence and outcomes, which extend beyond conventional radiographic parameters.

Previous studies have highlighted the importance of multiplanar correction, including first-metatarsal rotation, distal metatarsal articular angle (DMAA), and sesamoid position, as determinants of stability and recurrence; however, reported associations vary with study design and imaging protocols [25,26,27,28]. Our findings complement this evidence by showing that patients valued endpoints such as footwear autonomy and reduced anxiety, which only partially overlap with traditional alignment targets.

Within this context, the adjustable intramedullary T-plate fixation technique, which allows intraoperative adjustment of lateral translation, rotation, and DMAA before final fixation, provides a practical means of aligning radiographic correction with footwear-related function [16]. Previous reports on procedures emphasizing multiplanar correction have demonstrated both improved angular outcomes and favorable patient-reported results with acceptable complication rates [29]. The present qualitative findings extend this line of evidence by illustrating how patients perceived such refinements as contributing to everyday autonomy, for example, attending formal events without self-consciousness or selecting footwear based on preference rather than necessity. While the present findings are grounded in experiences following surgery with an adjustable intramedullary T-plate, the qualitative themes identified here may be transferable to other hallux valgus procedures that similarly achieve stable correction and permit a return to footwear aligned with occupational and social demands.

Another consistent message is the mismatch between commonly reported outcomes and patients’ own emphasis. Over the last decade, AOFAS and VAS scores have dominated the reporting of hallux valgus surgery; however, AOFAS is clinician-based and often fails to capture footwear tolerance, cosmetic ease, or role-specific participation. Even with improved radiographic alignment, dissatisfaction can persist when footwear restrictions or cosmetic concerns remain unresolved [8,30]. This underscores the need for patient-reported outcome measures that are sensitive to footwear characteristics and occupational demands, thereby making preoperative counselling more realistic and postoperative endpoints more meaningful to patients.

Clinically, the implications are direct. Preoperative counseling should pair functional targets with aesthetic and footwear goals, and co-design “rules of return” that balance heel height, event duration, and walking distance. Graduated exposure to formal footwear, rotation of pairs across the workweek, afternoon fitting to accommodate diurnal swelling, and prioritization of softer uppers or wider lasts can reduce scar pressure and anticipatory anxiety while preserving professional presentation. On the surgical side, emphasizing multiplanar correction and sesamoid reduction remains justified not only by recurrence-focused evidence [25] but also by the downstream likelihood of comfortable shoe choice, a patient-defined endpoint that repeatedly emerged in this study.

### Limitations

This study is an interpretivist qualitative inquiry employing purposive sampling, restricted to women in white-collar occupations at a single country/center; therefore, the transferability of the findings to manual/service occupations and to other cultural or climatic settings is limited. The reflexive thematic approach prioritizes depth over statistical generalizability, and inter-rater reliability was not calculated, consistent with contemporary reflexive thematic analysis frameworks. Nonetheless, credibility was strengthened through member checking, an audit trail, negative-case attention, and peer debriefing, although residual interviewer–participant dynamics and volunteer bias cannot be excluded. Interviews were conducted in Turkish and underwent translation/back-translation, which may introduce minor shifts in nuance despite efforts to ensure semantic accuracy.

## 5. Conclusions

This qualitative study shows that, among white-collar women who underwent corrective surgery for mild-to-moderate hallux valgus using adjustable intramedullary T-plate fixation, postoperative success was defined by functional improvement rather than radiographic correction alone. Participants described regaining autonomy in footwear, social ease, and professional confidence as the clearest markers of recovery. Across multiple interrelated themes and subthemes, participants framed recovery through expectations and motivations, postoperative physical experience, aesthetic perception, psychological responses, social and domestic support, and footwear identity and adaptation. These patient experiences align with the technical rationale of the procedure, which allows multiplanar adjustment to achieve stable realignment while supporting comfortable footwear use. The findings, therefore, complement previously reported quantitative outcomes of this technique by illustrating how radiographic and clinical gains translate into everyday mobility, identity, and participation. Integrating footwear expectations and occupational demands into preoperative counselling and postoperative guidance is essential to achieving outcomes that reflect patients’ priorities.

## Figures and Tables

**Table 1:** Demographic and occupational characteristics of the participants included in the qualitative study.

Number	Age (Years)	Occupation	Surgery–Interview Interval (Months)
1	38	Teacher	14
2	45	Office Administrator	16
3	40	Lawyer	18
4	35	Nurse (Hospital Administrative Role)	13
5	42	Finance Specialist	20
6	47	Engineer	17
7	44	Corporate Services Officer	15
8	39	Corporate Lawyer	21
9	51	Office Administrator	24
10	37	Human Resources Specialist	12
11	48	Healthcare Services Manager	18
12	43	Information Technology Specialist	16
13	36	University Lecturer/Academic Staff	13
14	46	Administrative Clerk	22
15	41	Architect	15
16	49	Project Coordinator	23
17	39	Teacher	14
18	52	Banker	24
19	42	Teacher	16
20	50	Government Officer—Ministry Employee	22
21	45	Executive Assistant	18
22	40	School Administrator/Principal	15
23	38	Accountant	13
24	43	Marketing Specialist	19
25	47	Teacher	21
26	44	Office Administrator	17
27	41	Lawyer	14

**Table 2:** Themes and subthemes related to the experiences of patients undergoing hallux valgus surgery.

Themes	Subthemes	Frequencies (Participants)
Expectations and Motivations Before Surgery	*Foot-related physical and cosmetic complaints*	25
	*Shoes, discomfort, and social appearance pressure*	24
	*Decision-making and expectations from surgery*	27
Postoperative Physical Experience	*Pain course, swelling, weight-bearing and wound care*	23
	*Changes in mobility and shoe choices*	22
	*New physical routines (footwear strategies, toe exercises)*	20
Aesthetic Perception and Body Image	*Perception of foot appearance after surgery*	26
	*Comfort in public settings*	24
	*Confidence, embarrassment, and identity-related shifts*	22
Psychological Process and Emotional Response	*Fear of recurrence or complications*	21
	*Satisfaction vs. disappointment*	25
	*Emotional adaptation and motivation for change*	20
Social and Domestic Support	*Family encouragement or discouragement pre-/post-op*	18
	*Impact on household roles and work life*	20
	*Partner/spouse opinions*	19
Footwear Identity and Adaptation Strategies	*Ability to wear preferred shoes*	24
	*Comfort versus appearance trade-offs*	22
	*Social expectations around footwear*	20
	*Adaptation via insoles, brands, and new habits*	21

## Data Availability

The data presented in this study are available on reasonable request from the corresponding author. The interview transcripts are not publicly available due to privacy and ethical restrictions, as they contain potentially identifiable personal information shared by participants during qualitative interviews.

## Supplementary Materials

The following supporting information can be downloaded at https://www.mdpi.com/article/10.3390/healthcare14040547/s1: Supplementary Material S1: Detailed Surgical Technique for Adjustable Intramedullary T-Plate Fixation; Supplementary Material S2: Qualitative Data Collection Materials, Form SA: Semi-Structured Interview Form, Form SB: Member Checking Form.
