# Supplementary material for: Footwear Identity and Postoperative Experiences of White-Collar Women After Hallux Valgus Surgery: A Qualitative Study
Source: Healthcare (Basel). 2026 Feb 22;14(4):547. doi: 10.3390/healthcare14040547 (PMC12941372; doi:10.3390/healthcare14040547)
Supplement: Supplementary file 1 [file healthcare-14-00547-s001.zip › Supplementary Material S1 (Surgical Technique).pdf]

Supplementary Material S1

Surgical Technique [16]

All operations were carried out by the same experienced surgical team following a standardized protocol. After induction of spinal anesthesia, a thigh tourniquet was applied. A medial incision was made adjacent to the first metatarsophalangeal (MTP) joint, and the capsule was opened longitudinally before being carefully elevated proximally.

The osteotomy level was defined with a K-wire placed tangential to the proximal sesamoids and perpendicular to the longitudinal axis of the first metatarsal. Under fluoroscopic control, a distal metatarsal osteotomy was created parallel to the K-wire to allow the passage of a microcutter. When the distal metatarsal articular angle (DMAA) was increased, a medial-based closing wedge was added to the osteotomy in order to reduce the DMAA toward neutral, and the bony wedge was excised.

The distal fragment was shifted laterally by hand. The medullary canal of the proximal segment was exposed, and a curved clamp was temporarily introduced intramedullary with the concave side facing medially, facilitating maximal lateral translation of the distal fragment. The clamp was then removed, and a pre-contoured intramedullary "T" miniplate was advanced into the proximal canal until two adjacent distal holes remained outside the bone.

Rotational alignment of the metatarsal head was assessed fluoroscopically. A joystick K-wire was placed into the metatarsal head to correct pronation as required. Two locking screws were subsequently inserted through the distal holes of the plate to secure fixation. Any step-off resulting from lateral translation was corrected with a motorized mini saw. The adequacy of lateralization was judged by the position of the sesamoids on dorsoplantar fluoroscopy. If displacement was sufficient, the system was locked with a distal locking screw; if further translation was necessary, a non-locking screw was applied and gradually tightened until the sesamoids were appropriately reduced. Sagittal alignment was also checked fluoroscopically.

In cases requiring additional correction, an Akin osteotomy of the proximal phalanx was performed and stabilized with a cannulated screw. A bone tunnel was created proximally with a K-wire, and the previously elevated capsule was reattached using sutures passed through this tunnel. After final fluoroscopic confirmation of alignment and fixation, the wound was closed in layers.
