# Supplementary material for: Footwear Identity and Postoperative Experiences of White-Collar Women After Hallux Valgus Surgery: A Qualitative Study
Source: Healthcare (Basel). 2026 Feb 22;14(4):547. doi: 10.3390/healthcare14040547 (PMC12941372; doi:10.3390/healthcare14040547)
Supplement: Supplementary file 1 [file healthcare-14-00547-s001.zip › Supplementary Material S2 (Forms A and B).pdf]

Form SA. Semi-Structured Interview Form

Interviewer :
Participant No :
Date :

Footwear identity and postoperative experiences of white-collar women after hallux valgus surgery: a qualitative study

Ethical Approval Code: Adana City Training and Research Hospital-16/704-21.08.2025

Topic	Questions	Exploratory questions (Take notes)	Extra Notes
Preoperative experiences and decision-making process	What was your experience prior to surgery when you were diagnosed with hallux valgus? What factors influenced your decision to undergo this surgery?		
Postoperative period, recovery and pain	What was your experience after the surgery?		
Footwear comfort and selection	How would you describe your footwear experience after surgery?		
Body image and aesthetics	How do you feel about the appearance of your foot after surgery?		
Quality of life and return to daily life	How did your quality of life change after surgery?		
Psychological and emotional experiences	How would you describe your psychological and emotional experiences after surgery?		
Social and family support	What were your experiences with social and family support in the postoperative period?		
Persistent problems and coping	Do you have any ongoing problems? How do you manage them?		
Future expectations and recommendations	What are your expectations for the future after the surgery? Would you recommend this procedure to someone with similar problems? Why?		

Semi-Structured Interview Form (with exploratory questions)

1. What was your experience prior to surgery when you were diagnosed with hallux valgus? What factors influenced your decision to undergo this surgery?
   • Before the operation, what was your foot like on a typical day? What bothered you the most?
   • Where did it hurt most — the big toe joint or the ball of the foot? Was there a bump on the side of the big toe? Any hard skin/corns? Any pain under the ball of the foot? Did your toes crowd or rub? What shoe problems did you have (tightness, rubbing, needing a wider size or a bigger number, difficulty with high-heeled shoes)? What activities were hardest (standing long, walking, stairs, sports)?
   • What footwear difficulties did you have (size/width changes, wide toe box search, high-heeled shoes)?
   • What influenced your decision to undergo this surgery (function, aesthetics/cosmetic concerns, work/social reasons, advice from others)?
   • What treatments had you tried before (insoles/physiotherapy/orthoses/injections) and with what effect?
   • What were your expectations from surgery?
2. What was your experience after the surgery?
   • How would you describe your pain after surgery? Did it change over time (first 2 weeks, first month, after 3 months)?
   • Which methods/medications did you use for pain control (analgesics, ice, elevation), and how effective were they?
   • How was early mobilization and weight-bearing? Did you use a postoperative shoe/orthosis?
   • Did you encounter any challenges or unexpected issues (wound care, swelling)? How did you cope with them?
3. How would you describe your footwear experience after surgery?
   • Which shoes are comfortable or difficult now (sneakers, flats, boots, high heels)?
   • Any change in size/width/brand/last after surgery?
   • Do you use insoles/functional orthoses? In which situations are they helpful?
   • How do workplace dress codes or long-standing periods affect your shoe choices?
4. How do you feel about the appearance of your foot after surgery?
   • How do you feel about the appearance of your foot after surgery? (toe alignment, scar, swelling)
   • Confidence in social settings (beach/pool, special events); comments from others; habits of concealment or photo-sharing.
5. How did your quality of life change after surgery?
   • Changes in daily activities (standing time, walking distance, stairs, house/occupational roles, return to sport/physical activity).

• *Are you able to do anything now that you previously could not?*

• *Overall impact of these changes on your life.*

6. How would you describe your psychological and emotional experiences after surgery?

• *Emotional course of recovery; moments of anxiety or stress (e.g., fear of recurrence or over-/under-correction).*

• *Motivation and coping strategies; sources of reassurance.*

• *How have your thoughts about the future changed over time (hope vs. concern)?*

7. What were your experiences with social and family support in the postoperative period?

• *What support did you receive from family or close contacts? How did this affect your recovery?*

• *Any changes in relationships at home? What do these changes mean to you?*

• *Interaction with other patients or online support groups; how it helped.*

8. Do you have any ongoing problems? How do you manage them?

• *Any ongoing problems (metatarsalgia/transfer pain, stiffness/limited 1st MTP range, numbness/paresthesia, footwear friction, scar sensitivity, recurrence concerns)?*

• *How do you manage them (insoles/orthoses, shoe modifications, physiotherapy/self-management)?*

9. What are your expectations for the future after the surgery? Would you recommend this procedure to someone with similar problems? Why?

• *Concerns or hopes for the future (long-term durability, return to specific activities, pregnancy plans if relevant, return to heels).*

• *Do you feel your lifestyle priorities have shifted?*

• *Would you recommend this procedure to someone with similar problems? Why?*

Form SB. Member Checking Form

Interviewer :
Participant No :
Date :

Footwear identity and postoperative experiences of white-collar women after hallux valgus surgery: a qualitative study

Ethical Approval Code: Adana City Training and Research Hospital-16/704-21.08.2025

Questions	Answers	Correction Notes
What was your experience prior to surgery when you were diagnosed with hallux valgus? What factors influenced your decision to undergo this surgery?	XXXXXXXXXXXXXXXXXXXXXXX<br>**Partıcıpant's Answers / Opınıons in the 1ST intervıew** XXXXXXXXXXXXXXXXXXXXXXX	
What was your experience after the surgery?	XXXXXXXXXXXXXXXXXXXXXXX<br>**Partıcıpant's Answers / Opınıons in the 1ST intervıew** XXXXXXXXXXXXXXXXXXXXXXX	
How would you describe your footwear experience after surgery?	XXXXXXXXXXXXXXXXXXXXXXX<br>**Partıcıpant's Answers / Opınıons in the 1ST intervıew** XXXXXXXXXXXXXXXXXXXXXXX	
How do you feel about the appearance of your foot after surgery?	XXXXXXXXXXXXXXXXXXXXXXX<br>**Partıcıpant's Answers / Opınıons in the 1ST intervıew** XXXXXXXXXXXXXXXXXXXXXXX	
How did your quality of life change after surgery?	XXXXXXXXXXXXXXXXXXXXXXX<br>**Partıcıpant's Answers / Opınıons in the 1ST intervıew** XXXXXXXXXXXXXXXXXXXXXXX	
How would you describe your psychological and emotional experiences after surgery?	XXXXXXXXXXXXXXXXXXXXXXX<br>**Partıcıpant's Answers / Opınıons in the 1ST intervıew** XXXXXXXXXXXXXXXXXXXXXXX	
What were your experiences with social and family support in the postoperative period?	XXXXXXXXXXXXXXXXXXXXXXX<br>**Partıcıpant's Answers / Opınıons in the 1ST intervıew** XXXXXXXXXXXXXXXXXXXXXXX	
Do you have any ongoing problems? How do you manage them?	XXXXXXXXXXXXXXXXXXXXXXX<br>**Partıcıpant's Answers / Opınıons in the 1ST intervıew** XXXXXXXXXXXXXXXXXXXXXXX	
What are your expectations for the future after the surgery? Would you recommend this procedure to someone with similar problems? Why?	XXXXXXXXXXXXXXXXXXXXXXX<br>**Partıcıpant's Answers / Opınıons in the 1ST intervıew** XXXXXXXXXXXXXXXXXXXXXXX	
